# Fluorescence Properties of a Novel Cyanobacteriochrome GAF Domain from *Spirulina* that Exhibits Moderate Dark Reversion

**DOI:** 10.3390/ijms19082253

**Published:** 2018-08-01

**Authors:** Xian-Jun Wu, Hong Yang, Yi Sheng, Yong-Li Zhu, Ping-Ping Li

**Affiliations:** 1 College of Biology and the Environment, Nanjing Forestry University, Nanjing 210037, China; yanghong0406108@163.com (H.Y.); shengyi_1993@163.com (Y.S.); lyly1262011@126.com (Y.-L.Z.); ppli@njfu.edu.cn (P.-P.L.); 2 Collaborative Innovation Center of Sustainable Forestry in Southern China of Jiangsu Province, Nanjing Forestry University, Nanjing 210037, China

**Keywords:** fluorescent proteins, cyanobacteriochrome, photoconversion, dark reversion

## Abstract

Cyanobacteriochromes (CBCRs) are biliproteins for photoreception that are present in cyanobacteria. These proteins possess one or more unique cGMP-specific phosphodiesterase/adenylate cyclase/FhlA (GAF) domains that can covalently bind the linear tetrapyrrole (bilin). Light absorption triggers the photoisomerization of bilin between the 15Z and 15E photostates. The 15E photoproduct of some CBCR GAF domains can revert to the stable 15Z state in the absence of light. In some cases, this property makes these domains function as sensors of light intensity or as red/dark optogenetic switches. However, there have been few reports regarding the applicability of these fluorescent properties. Here, we report a red/green cyanobacteriochrome GAF domain from *Spirulina subsalsa*, designated SPI1085g3, which exhibited photoconversion from the red-absorbing dark state (Pr, λmax = 642 nm) to the orange-absorbing photoproduct state (Po, λmax = 590 nm), and exhibited moderate dark reversion (*t*_1/2_ = 3.3 min) from the Po state to the Pr state. The SPI1085g3 Pr state exhibited intense red fluorescence (λmax = 662 nm), with a quantum yield of 0.14. The fluorescence was switched off by red light irradiation and increased in the dark. Replacement of Cys448 of SPI1085g3 with Ser resulted in a slightly improved fluorescence quantum yield and nearly 13-fold faster dark reversion (*t*_1/2_ = 15.2 s) than that of the wild type. This novel red/dark-switchable fluorescent biliprotein expands the present repertoire and diversity of photoswitchable fluorescent protein candidates.

## 1. Introduction

Cyanobacteriochromes (CBCRs) are photoreceptors that bind to a linear tetrapyrrole in cyanobacteria and are distantly related to red/far-red light-sensing phytochromes [1]. Only the cGMP-specific phosphodiesterase/adenylate cyclase/FhlA (GAF) domains of CBCRs are necessary for chromophore incorporation and proper photoconversion and respond to a wide light spectrum, ranging from the near-ultraviolet region to the red region of the visible spectrum [2,3,4,5,6,7,8,9,10,11,12,13,14,15,16]. On the other hand, most canonical phytochromes require a large unit, consisting of Per/Arnt/Sim (PAS), GAF, and phytochrome-specific (PHY) domains, to respond to red and far-red light [17,18]. CBCR GAF domains can utilize phycocyanobilin (PCB), phycoviolobilin (PVB), and biliverdin (BV) as chromophores to detect light. PCB-binding CBCR GAF domains sense relatively long wavelengths of light, ranging from the blue region to the far-red region [3,9,10,12,13,14], whereas PVB-binding CBCR GAF domains sense rather short wavelengths of light, ranging from the ultraviolet region to the green region [5,6,8,15,16]. Recently, Some CBCR GAF domains that incorporate BV were identified in the chlorophyll-d-producing cyanobacterium *Acaryochloris marina*, sensing long wavelengths of light close to the near-infrared window [4,19,20]. All bilin chromophores are derived from heme, which can be oxidatively cleaved by heme oxygenase (HO1) to BV [21], which is reduced to PCB in the presence of PCB:ferredoxin oxidoreductase (PcyA) [22]. In addition, some CBCR GAF domains are capable of autoisomerizing PCB to PVB [16].

The bilin chromophore is covalently attached via a thioether linkage to a conserved Cys of CBCR GAF domains, wherein photoexcitation triggers the reversible isomerization of the bilin 15,16-double bond. In the stable dark-adapted state, absorption of light leads to the formation of a metastable photoproduct, which can revert to the dark state over seconds to days via a process known as dark reversion [14,23,24,25,26]. The photophysical and photochemical behaviors of CBCR GAF domains are profoundly influenced by the chromophore microenvironment, leading to the remarkable spectral diversity of these domains, which is dependent on the chromophore species and primary sequence elements that they contain. Accordingly, CBCR GAF domains are categorized into various subfamilies with a wide variety of photocycles, such as red/green, blue/green, and green/red [8,9,10]. Among these CBCR GAF domains, red/green-type CBCR GAF domains are widely distributed among various cyanobacteria and are well characterized. These domains covalently bind PCB and reversibly photoconvert between a red-absorbing thermostable form (Pr) and a green-absorbing metastable form (Pg) [4,9,27,28].

Some red/green-type CBCR GAF domains exhibit red fluorescence in the dark-adapted state. When reversible photoisomerization occurs, the fluorescence can be correspondingly switched with red and green light, suggesting that the CBCR GAF domains are suitable for the development of photoswitchable fluorescent proteins [4,27]. In most cases, the red/green-type CBCR GAF domains exhibit a very slow process of dark reversion, with a half-life of many hours, and exhibit low-fluorescence quantum yields due to chromophore photoisomerization [29]. Moreover, NpR6024g5, a previously described red/green-type CBCR GAF domain, exhibited intense fluorescence, with a high fluorescence quantum yield close to 0.14, but had no detectable photoconversion [25]. Recently, additional red/green-type CBCR GAF domains have been shown to exhibit unidirectional photoconversion and rapid dark reversion and to even function as sensors of light intensity upon the occurrence of sufficiently rapid dark reversion [23,24,25,26]. Meanwhile, a red/dark reversible switch, with a half-life of 5.6 min and based on the PCB-binding red/green CBCR GAF domain AnPixJg2_DR6, was used to create a cAMP synthesis system, which detected a significant difference in adenylate cyclase activity under red light and under dark incubation conditions; another domain, AnPixJg2, with a half-life of 17.2 s, did not exhibit significant light-quality-dependent activity [23]. This finding implies that moderate dark reversion maybe is more suitable for the development of red/dark optogenetic tools than very fast dark reversion, but this remains to be proved.

Here, we report a red/green-type CBCR GAF domain from *Spirulina*, designated SPI1085g3, which exhibits not only moderate dark reversion but also intense red fluorescence, with a quantum yield close to that of NpR6024g5. The 15E photoproduct can revert back to the 15Z dark-adapted state with an increase in fluorescence. The C448S mutant exhibited a slightly improved fluorescence quantum yield and nearly 13-fold faster dark reversion than the wild type. This CBCR GAF domain, which exhibits intense red fluorescence with a red/dark switch, expands the present repertoire and diversity of photoswitchable fluorescent protein candidates.

## 2. Results

### 2.1. Sequence Characteristics of SPI1085g3

**Figure 1:**
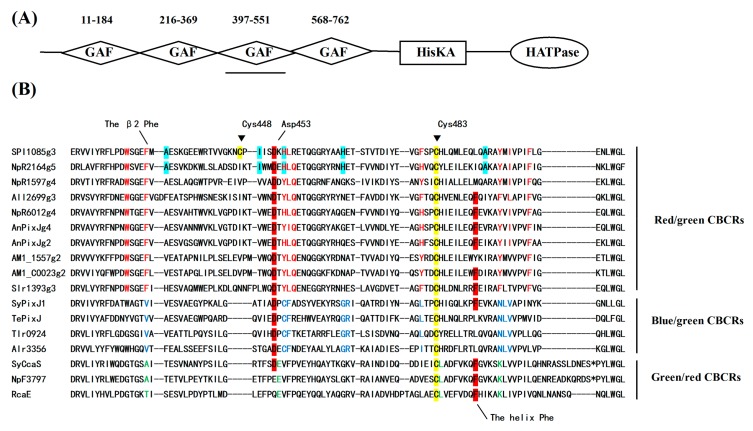
Domain and sequence analysis of cyanobacteriochromes (CBCRs). (**A**) Domain architecture of SPI9445_RS0121085 (SPI1085); (**B**) Sequence alignment of SPI1085g3 and other CBCR cGMP-specific phosphodiesterase/adenylate cyclase/FhlA (GAF) domain homologs. Residues conserved or enriched in red/green (red), green/red (green), and blue/green (blue) CBCR GAF domains are color-coded. The helix Phe and Asp motifs are highlighted in red. Cys448 and Cys483 in SPI1085g3 are highlighted in yellow. The residues highlighted in light green are presumed to play key roles in improving the fluorescence quantum yields of red/green CBCR GAF domains.

SPI9445_RS0121085 (abbreviated as SPI1085) is a hypothetical signal transduction protein from *Spirulina subsalsa* PCC 9445 that contains 1046 amino acid residues. SPI1085 is composed of four GAF domains, one His kinase (HK) domain, and one response regulator (RR) domain (Figure 1A). The third GAF domain (SPI1085g3, residues 397-551) is a red/green-type CBCR GAF domain according to our sequence alignment (Figure 1B). The sequence of this domain shares 67% residue identity with that of Slr1393g3 [14,27], and contains several characteristic amino acid substitutions that are highly conserved in red/green CBCRs. The DNA segment encoding SPI1085g3 was cloned from the *S. subsalsa* FACHB351 genome. The obtained amino acid sequence (Figure S1) shares 95% residue identity with the SPI1085g3 sequence of *S. subsalsa* PCC 9445.

### 2.2. SPI1085g3 from a PCB-Producing E. coli BL21 Strain

**Figure 2:**
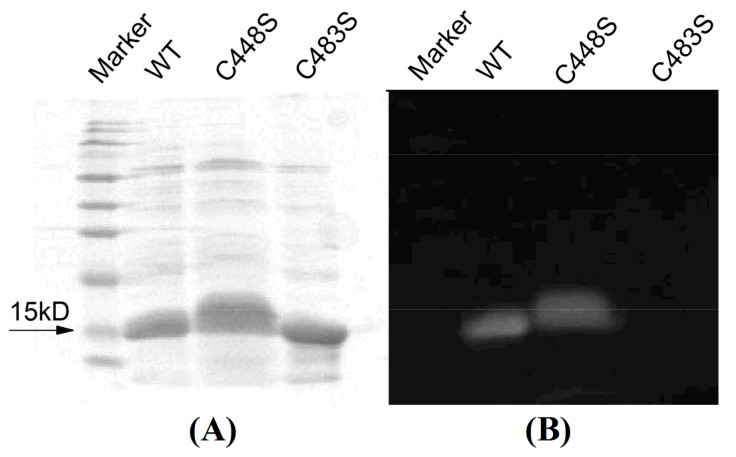
SDS-PAGE of SPI1085g3 (WT) and its variants (C448S and C483S) visualized by (**A**) Coomassie brilliant blue staining and (**B**) zinc-induced fluorescence. The marker (SMOBiO, PM2510) indicates protein molecular weights of 180, 140, 100, 75, 60, 45, 35, 25, 15, and 10 kD from top to bottom.

The plasmid carrying the gene coding for SPI1085g3 was transformed into an *E. coli* strain engineered to produce PCB. The relevant genes were coexpressed and the biliprotein was biosynthesized in *E. coli*. After induction with 0.1 M IPTG at 20 °C overnight, the cells exhibited a deep blue color. The His-tagged SPI1085g3 was purified by Ni^2+^-affinity chromatography. A band with a molecular weight of ~15 kD, which is consistent with the calculated molecular mass of His-tagged SPI1085g3, was observed by SDS-PAGE (Figure 2A, Wide Type, WT). In addition, this protein was covalently bound to a linear tetrapyrrole, judging from the band with strong zinc-dependent fluorescence observed by SDS-PAGE (Figure 2B, WT).

### 2.3. Photoconversion and Moderate Dark Reversion of SPI1085g3

**Figure 3:**
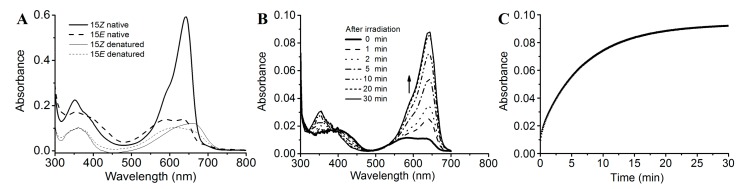
Photoconversion and dark reversion of SPI1085g3. (**A**) Absorbance spectra of native (heavy lines) and acid-urea denatured (thin lines) biliprotein. The dashed lines correspond to the 15E state obtained after irradiation with 653/20-nm light; (**B**) absorbance spectra acquired at the indicated times after irradiation; (**C**) absorbance at 642 nm monitored after driving the biliprotein to the E-state.

The PCB-binding SPI1085g3 exhibited a red-absorbing state (Pr) with an absorption peak at 642 nm and a Soret band at 352 nm. In addition, no other significant absorption peaks were observed (Figure 3A). However, when irradiated with red light, this biliprotein exhibited an orange-absorbing state (Po) and a reduction in red absorption, which is similar to the CBCR domains of the tandem sensor All2699 from Nostoc sp. PCC 7120 and NpR1597 from Nostoc punctiforme, which exhibited reversible red/orange photocycles (Figure 1B) [14,25]. Similar to the above mentioned CBCR domains, SPI1085g3 exhibited a mix of red and orange states in its Po form, exhibiting two absorption peaks at ~642 and ~590 nm, and seemed to undergo incomplete photoconversion or rapid dark reversion from the Pr state to the Po state. Furthermore, dark reversion from the orange-absorbing state to the red-absorbing state was observed (Figure 3B), indicating that the red-absorbing state is dark-adapted and the orange-absorbing state is the photoproduct. We think that the unidirectional photoconversion and dark reversion could be repeated many times without appreciable deterioration of the spectra, but this remains to be further proved. In most red/green CBCRs, the Pg photostate exhibits a maximal absorbance in the green region of the red/green photocycle. However, some conserved amino acid residues may be involved in tuning the color of the photoproducts [30,31] and could even be responsible for PCB modification [12].

We further measured the dark reversion kinetics of SPI1085g3 at the peak wavelength of 642 nm (Figure 3C). SPI1085g3 exhibited moderate dark reversion with a half-life of 3.3 min, which lies between the half-lives of All2699g3 (20 s) and NpR1597g4 (3.5 h). Some key amino residues are involved in photoproduct stability. For example, replacement at the Leu294/IIe660 positions of AnPixJg2 and AnPixJg4 greatly affected the dark reversion kinetics [23]. This moderate dark reversion provides a basis for the design of novel red/dark optogenetic tools. To conclusively identify the chromophore species and their configurations, SPI1085g3 was subjected to acid denaturation. The absorption spectra of the denatured proteins were obtained. The absorption maxima of denatured SPI1085g3 Pr and Po were observed at ~662 nm and ~604 nm, respectively (Figure 3A). Irradiation of denatured Po with white light resulted in conversion to the denatured Pr state, whereas irradiation of denatured Pr with white light did not result in a significant change (Figure S3). These results clearly showed that the Pr and Po states bound the unmodified PCB and exhibited typical 15Z/15E isomerization.

### 2.4. Intense Red Fluorescence of SPI1085g3

**Figure 4:**
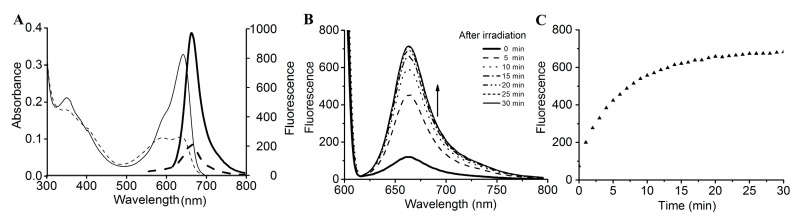
Fluorescence spectra and changes in intensity during dark reversion. (**A**) Absorbance (thin lines) and fluorescence (heavy lines) spectra of the 15Z (solid lines) and 15E (dashed lines) states; (**B**) fluorescence spectra acquired at the indicated times during dark reversion; excited at 600 nm; (**C**) fluorescence intensity change at 662 nm during dark reversion; excited at 600 nm.

The SPI1085g3 dark-adapted state exhibited intense fluorescence, with an emission maximum at 662 nm. However, after photoconversion from the Pr state to the Po state, the fluorescence of the Po state was barely detectable, with only a small amount of fluorescence detectable from the Pr state at an appropriate excitation wavelength (Figure 4A). SPI1085g3 Pr exhibited a markedly high fluorescence quantum yield of 0.14, which is comparable to that of several phycocyanins (Table 1) [32,33] and is similar to that of NpF2164g5, a red/green CBCR GAF domain with intense red fluorescence [25]. Interestingly, no photoconversion was detected in NpF2164g5, whereas significant photoconversion and dark reversion was observed in SPI1085g3. This result implied that there is no essential relationship between fluorescent intensity and photoconversion in such red/green CBCR GAF domains, and increased photoconversion did not lead to a decrease in the fluorescence of these domains. We reasoned that fluorescence and photoconversion could be attributed to different amino acid motifs (Figure 1B). In addition, we detected a change in fluorescent intensity during dark reversion (Figure 4B). The solution of SPI1085g3 was subjected to constant red light irradiation, resulting in the photoconversion of Pr to Po concomitant with a decrease in fluorescence to a level close to the background; however, the fluorescence was not completely abolished by red irradiation, which was possibly due to rapid dark reversion. In the dark, Po reverted to Pr concomitant with an increase in fluorescence. Furthermore, we measured the kinetics of fluorescence recovery when going from the Po photostate to the Pr photostate at 662 nm (Figure 4C). Fluorescence intensity gradually increased to a level close to that of the Pr state within 30 min when excited with 620 nm light, and this increase was identical in rate and extent to the increase in absorbance. This result indicated that the change in fluorescence intensity matched the change in absorbance. In this case, photoconversion and dark reversion could be repeated many times and will not affect the cyclic switching of fluorescence intensity.

### 2.5. Site-Directed Mutagenesis of SPI1085g3

**Figure 5:**
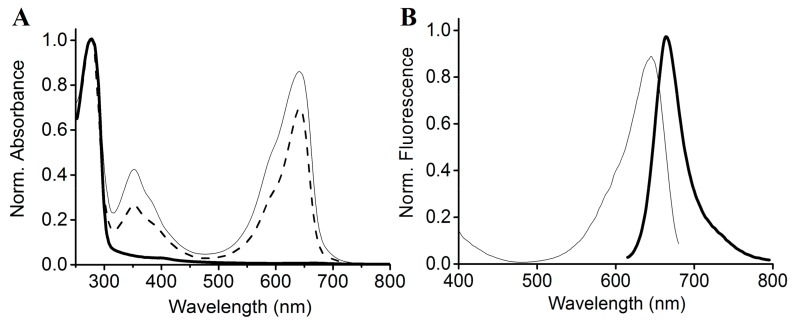
Absorbance and fluorescence spectra of the mutant SPI1085g3. (**A**) Absorbance spectra of SPI1085g3 (dashed line), SPI1085g3(C448S) (thin solid line), and SPI1085g3(C483S) (heavy solid line). Protein absorbance values at 280 nm were adjusted to be equal; (**B**) fluorescence excitation (thin line) and emission (solid line) spectra of SPI1085g3(C448S); excited at 600 nm.

According to our sequence alignment, SPI1085g3 has two cysteines, the unconserved Cys448 and the canonical Cys483 (Figure 1B). To show that Cys483 is covalently bound to the chromophore, we performed site-directed mutagenesis, substituting the Cys483 residue with a serine to generate SPI1085g3(C483S). The Zn blot of the mutant indicated that no PCB was bound to SPI1085g3(C483S) (Figure 2B). In addition, SPI1085g3(C483S) did not absorb an appreciable amount of visible light (Figure 5A). These results indicate that SPI1085g3 covalently binds PCB via Cys483. To understand the effects of Cys448 on bilin-binding, we prepared another mutant protein, namely, SPI1085g3(C448S). This replacement resulted in a significant improvement of the expression yield of the PCB-binding biliprotein (A_C448S,642 nm_ > A_WT,642 nm_) (Figure 5A), which indicates that the C448S mutant had higher chromophore-binding efficiency than the wild-type protein. This difference may be attributed to the effects of this replacement on the chromophore-binding pocket to facilitate protein expression and chromophore incorporation.

**Figure 6:**
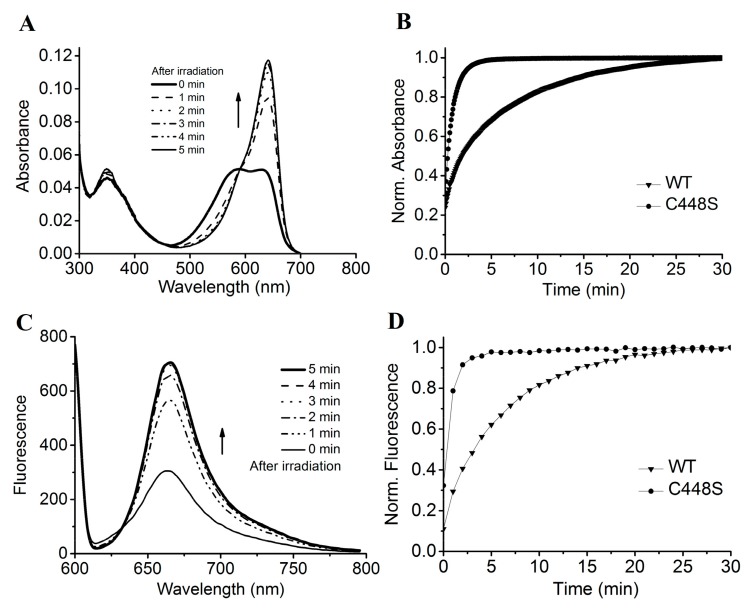
Dark reversion of SPI1085g3(C448S). (**A**) Absorbance spectra of SPI1085g3(C448S) acquired at the indicated times after irradiation; (**B**) normalized absorbance at 642 nm monitored after driving the biliprotein to the *E*-state. WT represents the wild-type SPI1085g3, and C448S represents SPI1085g3(C448S); (**C**) fluorescence spectra of SPI1085g3(C448S) acquired at the indicated times during dark reversion; excited at 600 nm; (**D**) normalized fluorescence intensity at 662 nm during dark reversion; excited at 600 nm. WT represents the wild-type SPI1085g3, and C448S represents SPI1085g3(C448S).

The C448S mutant exhibited unidirectional photoconversion from the Pr (absorbance maximum, 642 nm) to Po (absorbance maximum, 590 nm) state and rapid dark reversion from Po to Pr (Figure 6A). Furthermore, we measured the dark reversion kinetics of the mutant from the Po to Pr state (Figure 6B). The half-life of the mutant was estimated to be 15.5 s by monitoring the increase in absorbance at 662 nm in dark, which was approximately 13-fold faster than that of the wild type (Table 1). The Cys448 residue of SPI1085g3 is likely to be a key residue for the stability of the Po form. The half-life of SPI1085g3(C448S) is very similar to that of some red/green CBCR GAF domains, such as NpF164g7 and AnPixJg4, demonstrating very fast dark reversion to function as a sensor of light intensity. This result suggests that SPI1085g3(C448S) is also likely to function as a power sensor.

The PCB-binding SPI1085g3(C448S) exhibited intense red fluorescence of the Pr form at approximately 662 nm, with a quantum yield comparable to that of the wild-type protein (Figure 5B, Table 1). The Pr fluorescence of SPI1085g3(C448S) could be switched off by illumination with red light followed by a fast recovery in the dark (Figure 6C). The fluorescence intensity of this mutant increased much faster than that of the wild-type protein (Figure 6D), corresponding to an increase in the Pr absorbance during dark reversion. This is the first time that a rapid change in fluorescence intensity during such a rapid dark reversion has been reported.

## 3. Discussion

In this study, we prepared a typical PCB-binding red/green CBCR GAF domain, namely, SPI1085g3, which not only exhibited unidirectional photoconversion and moderate dark reversion from the orange-absorbing photoproduct to the red-absorbing dark state but also exhibited intense red fluorescence in the dark state with a markedly high fluorescence quantum yield. The fluorescence could be switched off by red light and recovered in the dark. This property makes SPI1085g3 a novel red/dark switch that could be developed as an unusual photocontrollable fluorescent protein.

SPI1085g3 exhibited a red-absorbing 15Z Pr state similar to that of most of red/green CBCR GAF domains but a red-shifted orange-absorbing 15E Po state rather than a green-absorbing one. The red-shifted photoproduct has been reported previously for All2699g3 and NpR1594g4 [14,25]. In these cases, the spectrum of the photoproduct overlaps with that of the 15Z dark state, leading to potential challenges with the use of difference spectroscopy. However, a distinct absorption peak of SPI1085g3 at 590 nm was observed upon sufficient photoconversion and by rapid detection. Furthermore, the 15E-PCB chromophore of the photoproduct was confirmed by the acid-denaturation assay. The redshift of the photoproduct was attributed to the influence of the protein on the spectral properties of the chromophore. Previous studies have supported a trapped-twist mechanism for spectral tuning of red/green CBCR photoproducts [30], where a pair of conserved Phe residues (the β2 Phe and the helix Phe) is part of a “steric blockade” that stabilizes the twisted geometry of the 15E photoproduct. When lacking the Phe residues, red-shifted photoproducts of red/green CBCRs may occur. SPI1085g3 possesses the β2 Phe but lacks the helix Phe, which has been replaced with Leu (Figure 1B), which may explain the presence of the red-shifted photoproduct. Moreover, we surveyed the amino acid residues equivalent to the β2 Phe and helix Phe in AM1_1557g2, NpF2164g5, and NpR1594g4 [4,25] and found that these domains have the β2 Phe residue but lack the helix Phe residue (Figure 1B). This finding strongly supports the importance of the helix Phe for tuning of the red-shifted photoproduct in most red/green CBCR GAF domains (Figure 1B).

SPI1085g3 exhibited moderate dark reversion from the Po state to the Pr state. To date, red/green CBCR GAF domains have been found to exhibit a broad range of dark reversion rates, with half-lives ranging from seconds to days [23,25,26]. The structural basis for the variety of dark reversion rates in CBCRs is not completely clear. Recent research has identified some key amino acid residues for rapid dark reversion in AnPixJg4 by comparative analysis of amino acid sequences [23]. However, the equivalent residues in some other red/green CBCR GAF domains with equally fast rates of dark reversion were different. In this study, The C448S mutant exhibited a spectrum that was almost identical to that of SPI1085g3, but unexpectedly, this mutant underwent a nearly 13-fold faster dark reversion than SPI1085g3. This result suggested a significant effect of Cys448 on the dark reversion kinetics of SPI1085g3, but Cys448 is not a conserved residue and has not been discovered at the equivalent positions of other red/green CBCR GAF domains. These results indicate that slight differences rather than structural differences were responsible for the dark reversion. Moreover, Cys448 of SPI1085g3 is located five residues upstream of the Asp492 residue, which is positioned toward the D-ring in the 15E isomer, based on the structural information for TePixJ [34,35]. This structural arrangement facilitates the photoproduct stability. Therefore, slight differences between some amino acids flanking the equivalent Asp492 may result in distinctive effects on dark reversion. Here, we could roughly categorize the red/green CBCR GAF domains into three types according to their rates of dark reversion: very fast, very slow, and moderate. Red/green CBCR GAF domains of the first type, exhibiting very fast dark reversion, with half-lives of seconds, likely serve as power sensors but not as color sensors, for example, All2699g3, AnPixJg4 and the C448S mutant of SPI1085g3 in this study. Domains of the second type, exhibiting very slow dark reversion, with half-lives of hours, serve as red/green color sensors, such as Slr1393g3, AnPixJg2, and NpR6012g4 [9,28,31]. Domains of the third type, exhibiting moderate dark reversion, with half-lives of minutes, may be developed as unusual red/dark switches for optogenetic or/and in vivo imaging applications, for example, SPI1085g3 in this study.

Both SPI1085g3 and its C448S mutant exhibited intense red fluorescence in their Pr state, with a high quantum yield that was comparable to that of NpF2164g5, which did not exhibit any photoconversion [25]. These GAF domains all belong to the red/green subfamily, as defined by protein sequence, and exhibit conserved sequence motifs with high homology. Comparative analysis of the amino acid sequences of the GAF domains indicate that there are some key residues involved in the improvement of the fluorescence quantum yields of red/green-type CBCR GAF domains. Further work is needed to identify these residues. Conversely, these key amino acids also reflect the mechanisms that determine the fluorescence intensity. Moreover, SPI1085g3 exhibited both photoconversion and significantly intense fluorescence, indicating that improvement of the fluorescence quantum yield does not necessarily accompany a loss of photoconversion, although previous studies have indicated that enhanced fluorescence is correlated with decreased photoconversion efficiency [29]. In other words, it is possible to improve the fluorescence quantum yields of GAF domains as well as to retain the photoconversion properties of these domains. This finding is of great significance with regard to the engineering of bright CBCR-GAF-based photoswitchable fluorescent proteins for in vivo imaging.

SPI1085g3 exhibited unidirectional photoconversion from the 15Z Pr to 15E Po states with a decrease in fluorescence intensity, and in the dark, this domain reverted to the 15Z Pr state with an increase in the fluorescence intensity. We examined the fluorescence kinetics of SPI1085g3 during dark reversion. The increase in red fluorescence was consistent with the increase in Pr absorbance. This result suggests that when SPI1085g3 exhibited a red/orange photocycle that was dependent only on red light, the switching mode of the Pr fluorescence transformed from the expected red/orange switch to a unique red/dark switch. This is the first report of a novel switch that demonstrates the photochemical diversity of CBCR GAF domains and expands the repertoire of photocontrollable fluorescent proteins, providing a basis for in vivo imaging applications.

## 4. Materials and Methods

### 4.1. Strains and DNA Extraction

Live *S. subsalsa* FACHB351 was obtained from the Freshwater Algae Culture Collection of the Institute of Hydrobiology, Chinese Academy of Sciences (Wuhan, China). The alga was cultured in SP medium at 25 °C. The light intensity was 50 µmol m^−2^ s^−1^, with a 12-h light/12-h dark photoperiod cycle. The filaments were collected by centrifugation (5000 rpm, 2739× *g*) at the log phase. The *Spirulina* genomic DNA was extracted using TRIzol agent (Invitrogen, Carlsbad, CA, USA) according to the standard method recommended by the manufacturer.

### 4.2. PCR Amplification and Plasmid Construction

The SPI1085g3 (encoding the apoprotein SPI1085g3) and ho1 (encoding the heme oxygenase HO1) genes were amplified from *S. subsalsa* FACHB351 genomic DNA by polymerase chain reaction (PCR) using Pfu DNA polymerase (Tiangen Biotech, Beijing, China). The sequences for primer design were obtained from the GenBank nucleotide sequence database (accession No. WP_017306776 for SPI1085g3 and accession No. WP_017304095 for ho1). The primers used to amplify the SPI1085g3 gene were as follows: forward primer, 5′-TTC CGA GCT CA ATC CGA CGT TCT TTA AA CC-3′; reverse primer, 5′-GCG AAG CTT TAT GAT TGT GCC TGC ATT TGT-3′. The primers used to amplify the ho1 gene were as follows: forward primer, 5′-TC GCC ATG GGT GTT AGT TTA GCA GAA G T-3′; reverse primer, 5′-TAT GAA TTC TTC GGC GGT GGC TAA TTC GC-3′. The SPI1085g3 product was digested with the restriction enzymes Sac I and Hind III and inserted into Sac I- and Hind III-digested pETDuet-1 (Novagen, Darmstadt, Germany) to obtain the plasmid pETDuet-SPI1085g3. The ho1 gene was digested with the restriction enzymes Nde I and Bgl II and inserted into pETDuet-SPI1085g3 digested with the same enzymes, yielding the plasmid pETDuet-SPI1085g3-ho1. The pcyA gene (encoding PcyA), flanked by the restriction enzymes Bgl II and Xho I, was synthesized by GenScript based on the sequence from the GenBank nucleotide sequence database (accession No. WP017303809). The pcyA gene was then inserted into Bgl II- and Xho I-digested pETDuet-SPI1085g3-ho1, fused to the C terminus of ho1, yielding the plasmid pETDuet-SPI1085g3-ho1::pcyA. This plasmid was used as the template for site-directed mutagenesis of SPI1085g3, which was performed with appropriate primers using the Fast Site-Directed Mutagenesis Kit (Tiangen Biotech, Beijing, China) according to the manufacturer’s protocol. pETDuet-SPI1085g3(C448S)-ho1::pcyA was generated using the primers 5′-T GTG GTG GGG AAA AAC TCC CCA ATT ATT TCG-3′ (forward primer) and 5′-GA GTT TTT CCC CAC CAC AGT CCG CCA TTC TT-3′ (reverse primer). pETDuet-SPI1085g3(C483S)-ho1::pcyA was generated using the primers 5′-G GTG GGT TTT TCC CCT TCT CAT CTC CAA ATG-3′ (forward primer) and 5′-GA AGG GGA AAA ACC CAC CTC ATA AAT ATC CG-3′ (reverse primer). All primers were synthesized by GenScript. The sequences of the genes encoding SPI1085g3, HO1, PcyA, SPI1085g3(C448S) and SPI1085g3(C483S) were verified by DNA sequencing. The obtained nucleotide and amino acid sequences of the heme oxygenase HO1 from *S. subsalsa* FACHB351 are shown in Figure S2.

### 4.3. Expression and Purification of SPI1085g3

The three plasmids pETDuet-SPI1085g3-ho1::pcyA, pETDuet-SPI1085g3(C448S)-ho1::pcyA, and pETDuet-SPI1085g3(C483S)-ho1::pcyA were transformed into *E. coli* BL21(DE3) separately. The transformed cells were cultured at 18 °C in 100 mL Luria-Bertani (LB) medium supplemented with ampicillin (20 μg·mL^−1^) and chloromycetin (17 μg·mL^−1^). The cells were kept in an ice bath for 30 min after the OD_600_ reached 0.5. After induction with isopropyl β-d-thiogalactoside (1 mM) for 12 h, the cells were centrifuged at 12,000× *g* for 5 min at 4 °C. The cell pellet was resuspended in 5 mL ice-cold potassium phosphate buffer (KPB, 20 mM, pH 7.0) containing 0.5 M NaCl and disrupted by sonication for 5 min at 200 W (JY92-IIN, Scientz Biotechnology, Ningbo, China). The suspension was centrifuged at 12,000× *g* for 15 min at 4 °C, and the supernatant was purified via Ni^2+^-affinity chromatography on chelating Sepharose (Amersham Biosciences, Uppsala, Sweden) equilibrated with KPB-containing 0.5 M NaCl. The bound proteins remaining on the column were eluted with 1 mL saline KPB, containing, in addition, imidazole (0.5 M). After collection, the protein sample was dialyzed twice against the saline KPB [33].

### 4.4. SDS-PAGE and Zn-Induced Fluorescence Assay

A total of 50 μL of protein samples were analyzed by polyacrylamide gel electrophoresis (PAGE) in the presence of SDS. The SDS-PAGE gel was composed of a 10% resolving gel and a 5% stacking gel. The samples were boiled with 2×SDS sample buffer containing 30 mM β-mercaptoethanol for 5 min. The purified proteins were visualized by staining with Coomassie blue and the bilins in the samples were detected by Zn^2+^-induced fluorescence. Fluorescence was visualized through a 630-nm filter upon excitation at 530 nm (GenoSens1850, Clinx, Shanghai, China).

### 4.5. Spectroscopy

All experiments were conducted at room temperature. Red light was provided by a cold fiberoptic light source with a 150 W halogen lamp (Bocheng, Nanjing, China) through a bandpass filter with a peak wavelength of 653 nm and a 20-nm half-bandwidth (Rayan, Changchun, China). Light intensity at the sample plane for photoconversion was 15 μmol m^−2^ s^−1^. Samples were irradiated for 3 min for photoconversion.

Absorbance spectra were obtained on a PerkinElmer Lambda 365 spectrophotometer. Scanning kinetics during dark reversion were recorded at intervals of 1 min for 30 min within a wavelength range of 300–700 nm. Dark reversion kinetics were recorded at a wavelength of 642 nm with intervals of 5 s for 30 min. After denaturing the proteins in 8 M urea at pH 2.0 in the dark, the absorption spectra of the proteins were recorded. Then, the protein samples were irradiated with white light for 3 min, and the absorption spectra were recorded again.

Fluorescence spectra were recorded with a model LS 55 spectrofluorimeter (Perkin Elmer, Waltham, MA, USA). The excitation wavelength was 600 nm. Excitation and emission slits were set at 10 nm for all measurements, and the scan speed was 1200 nm/min. Fluorescence scanning kinetics during dark reversion were recorded at intervals of 5 min for 30 min. Fluorescence recovery kinetics at 662 nm were obtained at intervals of 1 min for 30 min. The spectra were recorded with a 0.1 s integration time, and the lamp was automatically turned off during intervals.

## 5. Conclusions

We isolated the PCB-binding CBCR GAF domain SPI1085g3, which exhibited reversible unidirectional photoconversion from the Pr state to the Po state and moderate dark reversion from the Po state to the Pr state. SPI1085g3 exhibited intense red fluorescence at 662 nm with a high fluorescence quantum yield. The fluorescence could be switched off by red light irradiation, and fluorescence recovery was observed after irradiation, which corresponded to the process of dark reversion to the Pr state. Substitution of Cys448 with a serine in SPI1085g3 led to a slightly improved fluorescence quantum yield and very fast dark reversion. These results indicate that SPI1085g3, as an unusual red/dark switch, can be used in optogenetics and as an imaging tool.

## Figures and Tables

**Table 1:** Spectral properties and dark reversion rates of reconstituted biliproteins.

Biliprotein	Absorbance				Fluorescence			Half-Life ^a^
	λ_max_ [nm]		ε [M^−1^·cm^−1^] × 10^5^		λ_max_ [nm]		*Φ*_F_	*t*_1/2_
	15Z	15E	15Z	15E	15Z	15E	15Z	15E to 15Z
SPI1085g3	642	590	0.98 ± 0.05	0.34 ± 0.02	662	−	0.14 ± 0.02	3.3 min
SPI1085g3(C448S)	642	590	0.96 ± 0.06	0.31 ± 0.03	662	−	0.15 ± 0.03	15.3 s

Spectra were obtained in potassium phosphate buffer (20 mM, pH 7.0). Extinction coefficients and fluorescence yields were averages of two independent experiments. Dark reversion was measured at room temperature by incubating the 15E photostate in the dark and measuring the absorbance at 642 nm. ^a^ The half-life was calculated by exponential fitting.

## Supplementary Materials

Supplementary materials can be found at http://www.mdpi.com/1422-0067/19/8/2253/s1.
