# Supplementary material for: Fluorescence Properties of a Novel Cyanobacteriochrome GAF Domain from Spirulina that Exhibits Moderate Dark Reversion
Source: Int J Mol Sci. 2018 Aug 1;19(8):2253. doi: 10.3390/ijms19082253 (PMC6121604; doi:10.3390/ijms19082253)
Supplement: Supplementary file 1 [file ijms-19-02253-s001.pdf]

Supplementary

Fluorescence Properties of a Novel Cyanobacteriochrome GAF Domain from *Spirulina* that Exhibits Moderate Dark Reversion

Xian-Jun Wu [1,2,*], Hong Yang [1], Yi Sheng [1], Yong-Li Zhu [1,2], and Ping-Ping Li [1,2]

[1] College of Biology and the Environment, Nanjing Forestry University, Nanjing 210037, China; yanghong0406108@163.com (H.Y.); shengyi_1993@163.com (Y.S.); lyly1262011@126.com (Y.-L.Z.); ppli@njfu.edu.cn (P.-P.L.)

[2] Collaborative Innovation Center of Sustainable Forestry in Southern China of Jiangsu Province, Nanjing Forestry University, Nanjing 210037, China

* Correspondence: xjwu@njfu.edu.cn; tel.: +86-25-8542-7210

```
1173   ATCCGACGTTCTTTAAACCTGCAAACCATTTTTAACACGGCGACTCAAGAAGTGCGGCAG
392    I  R  R  S  L  N  L  Q  T  I  F  N  T  A  T  Q  E  V  R  Q

1233   GTTTTACAAGCGGAACGGGTGGTGATTTACCGCTTTCTGCCGGATTGGAGCGGGGAATTT
412    V  L  Q  A  E  R  V  V  I  Y  R  F  L  P  D  W  S  G  E  F

1293   ATGGCGGAGTCCAAAGGGGAAGAATGGCGGACTGTGGTGGGGAAAAACTGCCCAATTATT
432    M  A  E  S  K  G  E  E  W  R  T  V  V  G  K  N  C  P  I  I

1353   TCGGACAAACATTTGCGAGAAACTCAGGGGGGTCGCTATGCTGCCCATGAAACCTCAACA
452    S  D  K  H  L  R  E  T  Q  G  G  R  Y  A  A  H  E  T  S  T

1413   GTTACGGATATTTATGAGGTGGGTTTTTCCCCTTGTCATCTCCAAATGTTAGAACAATTA
472    V  T  D  I  Y  E  V  G  F  S  P  C  H  L  Q  M  L  E  Q  L

1473   CAAGCCCGGGCTTATATGATTGTTCCCATTTTCTTGGGGGAGAATCTCTGGGGCTTGTTA
492    Q  A  R  A  Y  M  I  V  P  I  F  L  G  E  N  L  W  G  L  L

1533   GCGGCCTATCAAAATTCGGCCCCTCGTTACTGGCAAGCTGATGAGGTGGAACTGTTGACC
512    A  A  Y  Q  N  S  A  P  R  Y  W  Q  A  D  E  V  E  L  L  T

1593   CAAATTGGTTCTCAGTTGGGGATGGCCATTCAACAGGGGCAATATTTGCAACAAATGCAG
532    Q  I  G  S  Q  L  G  M  A  I  Q  Q  G  Q  Y  L  Q  Q  M  Q

1653   GCACAATCA

552    A  Q  S
```

Figure S1. Nucleotide and amino acid sequence of SPI1085(GAF3, 397-552aa) from *Spirulina subsalsa* FACHB351. The conserved cysteine-483-binding site is underlined with a solid line; the unconserved cysteine-448 residue is underlined with a wavy line, and boxes show the conserved motifs in CBCR GAF domains.

1 ATGGGTGTTAGTTTAGCAGAAGTGAATTTAGCCACAATGCTACGGGAAGGGACGAAAAAA
1 M G V S L A E V N L A T M L R E G T K K

61 TCCCATACTATGGCGGAAAATATGGGGTTTATTAAATGTTTCCTCAAAGGCGTAGTCGAA
21 S H T M A E N M G F I K C F L K G V V E

121 AAAAATTCCTACCGTAGATTAGCGGCTAATCTCTACTATGTTTATGGGGCGATGGAAGAA
41 K N S Y R R L A A N L Y Y V Y G A M E E

181 GAAATGGAACGCCTCAAAGATCATCCCATTTTGTCTAAGGTCTATTTCCCTGAATTAAAT
61 E M E R L K D H P I L S K V Y F P E L N

241 CGGAAAGCTAATTTAGAGCGGGATTTGTTCTATTACTACGGAGCAAACTGGCGGGAGGAA
81 R K A N L E R D L F Y Y Y G A N W R E E

301 ATTGAACTTTCTCCTACAGGTCAAGCCTATGTTAATCGCATTCATCATGTGGCGAATGAA
101 I E L S P T G Q A Y V N R I H H V A N E

361 AAGCCAGAACTCTTGGTGGCACATCTTTACACCCGCTATCTGGGGGATCTCTCTGGGGGA
121 K P E L L V A H L Y T R Y L G D L S G G

421 CAAATCCTCAAGAAAATTGCTCAGACGGCGATGAATCTTTCTGAGGGTGAAGGGACGGCT
141 Q I L K K I A Q T A M N L S E G E G T A

481 TTTTATCAGTTTGAGCAGATTCCCGATGAGAAGGCGTTCAAAACCCAATATCGGGCTGCA
161 F Y Q F E Q I P D E K A F K T Q Y R A A

541 ATGGACAGTTTACCTGTGGATCAACAAACGGCCGAGGCTATTGTTGATGAAGCCAATGAT
181 M D S L P V D Q Q T A E A I V D E A N D

601 GCGTTCGGCATGAATATGAAAATGTTCGGAGAATTAGAAGGGGAATTTGATCAAAGCTAT
201 A F G M N M K M F G E L E G E F D Q S Y

661 TGGTCAATGCTGTTTAATTCTTTAACTCGTCGTCGCAGTCGTGGGAGTACCGATGGCGAA
221 W S M L F N S L T R R R S R G S T D G E

721 TTAGCCACCGCCGAATAG
241 L A T A E *

Figure S2. Nucleotide and amino acid sequence of heme oxygenase 1 (HO1) from *Spirulina subsalsa* FACHB351. Underline indicates the ATG start codon and the TAG stop codon, and boxes show the conserved active sites.

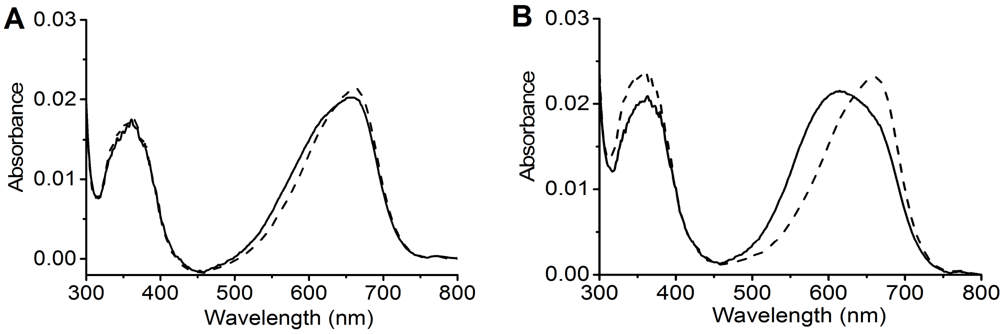

Figure S3. Acid denaturation of SPI1085g3. (A) Absorbance spectra of acid-denatured SPI1085g3 Pr state: absorbance spectra just after denaturation (solid line) and after while light illumination (dashed line). (B) Absorbance spectra of acid-denatured SPI1085g3 Pg state: absorbance spectra just after denaturation (solid line) and after while light illumination (dashed line).
